# Electrophysiological Phenotyping of hiPSC-Derived Atrial Cardiomyocytes Using Automated Patch-Clamp: A Platform for Studying Atrial Inherited Arrhythmias

**DOI:** 10.3390/cells14241941

**Published:** 2025-12-06

**Authors:** Verónica Jiménez-Sábado, Hosna Babini, Peter C. Ruben, Eric A. Accili, Thomas W. Claydon, Leif Hove-Madsen, Glen F. Tibbits

**Affiliations:** 1 Cellular and Regenerative Medicine Centre, BC Children’s Hospital Research Institute, Vancouver, BC V5Z 4H4, Canada; vjimenezs@santpau.cat (V.J.-S.); hosna_babini@sfu.ca (H.B.); 2 Biomedical Physiology and Kinesiology, Simon Fraser University, Burnaby, BC V5A 1S6, Canada; pruben@sfu.ca (P.C.R.); thomas_claydon@sfu.ca (T.W.C.); 3 Institut de Recerca de Sant Pau (IR SANT PAU), and CIBERCV, Hospital de la Santa Creu i Sant Pau, 08025 Barcelona, Spain; leif.hove@iibb.csic.es; 4 Department of Cellular and Physiological Sciences, University of British Columbia, Vancouver, BC V6T 1Z3, Canada; eric.accili@ubc.ca; 5 Instituto de Investigaciones Biomédicas de Barcelona (IIBB-CSIC), 08036 Barcelona, Spain; 6 Molecular Biology and Biochemistry, Simon Fraser University, Burnaby, BC V5A 1S6, Canada; 7 School of Biomedical Engineering, University of British Columbia, Vancouver, BC V6T 2B9, Canada

**Keywords:** automated patch-clamp, cardiac ion channels, human-induced pluripotent stem cell-derived atrial cardiomyocytes (hiPSC-aCMs), arrhythmia

## Abstract

**Highlights:**

**What are the main findings?**

- Developed an optimized dissociation and recording protocol enabling reliable automated patch-clamp recordings of major atrial ionic currents (I_Na_, I_CaL_, I_to_, I_Kur_, I_SK_, and I_f_) in hiPSC-derived atrial cardiomyocytes.
- Demonstrated that current profiles obtained with the automated Patchliner system resemble those of native human atrial cardiomyocytes, validating the physiological relevance of the model.

**What are the implications of the main findings?**

- The optimized automated patch-clamp workflow provides a robust platform for the functional characterization of ion channels and genetic variants implicated in atrial arrhythmias.
- This approach facilitates precision medicine applications and targeted drug development for atrial channelopathies.

**Abstract:**

Human-induced pluripotent stem cell-derived cardiomyocytes (hiPSC-CMs) represent a robust platform for modelling inherited cardiac disorders. Comparative analysis of ion channel activity in patient-specific and isogenic control lines provides critical insights into the molecular mechanisms underlying channelopathies and arrhythmias. Atrial-specific hiPSC-CMs (hiPSC-aCMs) exhibit distinct electrophysiological properties governed by unique ion channel expression profiles, underscoring the need for optimized methodologies to record atrial ionic currents accurately. Here, we characterized the electrophysiological features of hiPSC-aCMs using the Nanion Patchliner automated patch-clamp system. An optimized cell dissociation protocol was developed to enhance cell integrity and seal formation, while tailored intra- and extracellular solutions were employed to isolate specific ionic currents. Using this approach, we reliably recorded major atrial currents, including the sodium current (I_Na_), L-type calcium current (I_CaL_), transient outward potassium current (I_to_), ultrarapid component of the delayed rectifier current (I_Kur_), small-conductance calcium-activated potassium current (I_SK_), and pacemaker funny current (I_f_). The resulting current profiles were reproducible and consistent with those observed in native atrial cardiomyocytes. These findings establish the feasibility of the automated electrophysiological characterization of ion channels in hiPSC-aCMs. This platform enables more efficient investigation of pathogenic variants and facilitates the development of targeted therapeutics for atrial arrhythmias and related channelopathies.

## 1. Introduction

In the past decade, human-induced pluripotent stem cell-derived cardiomyocytes (hiPSC-CMs) have emerged as a promising model for studying cardiac diseases and drug screening [1,2,3,4,5,6]. Although differentiation efficiency has improved notably, the obtained cardiomyocytes are still immature compared to native human adult cardiomyocytes [7]. Moreover, some variability has been shown between laboratories and cell lines. Despite these challenges, the hiPSC-CM model is powerful as it presents different advantages to modelling human cardiac disease compared to animal models, which show notable differences in physiology and cellular regulation [8,9].

The hiPSC-CM model allows for the generation of chamber-specific cardiomyocytes using different protocols [5,10,11], which is an important advantage for modelling specific heart diseases and developing novel therapies to advance precision medicine. Moreover, the advancement of genome editing technology using CRISPR/Cas9 and other systems has allowed the creation of genetically engineered cell lines harbouring different genetic risk variants associated with inherited arrhythmic syndromes and their isogenic control lines. In line with this, hiPSC-CMs have been successfully used as models to study different arrhythmic diseases such as long QT syndrome (LQTS) [12,13], Brugada syndrome [14], catecholaminergic polymorphic ventricular tachycardia (CPVT) [15,16], Timothy syndrome [17], and atrial fibrillation (AF) [18,19].

Inherited arrhythmic syndromes usually involve abnormalities in cardiac ion channels or proteins associated with ion channels [20]. To assess the functionality of the ion channels for this disease group, the patch-clamp technique remains the gold standard for in vitro electrophysiology experiments. However, it presents different challenges as it is a low-throughput complex technique even with a highly experienced experimentalist [21]. Thus, there has been a high interest in developing automated patch-clamp technology to address the previously mentioned challenges [22,23,24,25]. Although automated patch-clamp technology presents some limitations, such as less consistency in high-quality spatial and temporal voltage control, it has been used in different cells, including hiPSC-CMs [26,27,28]. However, most studies using automated patch-clamp technology have been performed in hiPSC-derived ventricular CMs (hiPSC-vCMs), whose channel expression differs from hiPSC-derived atrial CMs (hiPSC-aCMs). Moreover, although sodium (I_Na_) and L-type calcium currents (I_CaL_) have been successfully recorded in hiPSC-vCMs [29], the transient outward potassium current (I_to_) and the ultrarapid component of the delayed rectifier current (I_Kur_) recordings in hiPSC-CMs have exhibited variability [30,31,32], and further investigation is needed. Here, we aim to establish a set of protocols to characterize different ion currents in hiPSC-aCMs that allows for the use of automated patch-clamp technology to study the impact of genetic variants associated with atrial arrhythmias on ion currents.

## 2. Materials and Methods

### 2.1. Differentiation of hiPSC into Atrial Cardiomyocytes

**Figure 1:**
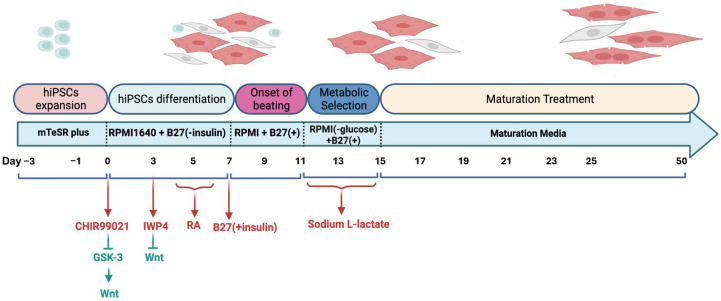
hiPSC-aCMs differentiation, metabolic selection, and maturation protocol. GSK-3, glycogen synthase kinase 3; RA, retinoic acid.

To study atrial arrhythmias, directed differentiation of hiPSCs into hiPSC-aCMs was performed using a protocol that has been previously refined and optimized [4,5]. The cell line was provided by Prof. Bjorn Knollman. As shown in Figure 1, hiPSCs were cultured in mTeSR Plus (05850, StemCell Technologies, Vancouver, BC, Canada) medium supplemented with a Rho-associated kinase (ROCK) inhibitor, Y-27632 dihydrochloride (1254, TOCRIS, Bio-Techne Canada, Toronto, ON, Canada), on Matrigel-coated substrates (356234, Corning, New York, NY, USA) (0.25 mg per 6-well plate, dissolved in DMEM/F-12 medium). hiPSCs were maintained in fresh mTeSR Plus medium for 3 days to achieve a confluent cell density between 70% and 90%. To initiate differentiation, hiPSCs were cultured in Roswell Park Memorial Institute Medium (RPMI) 1640 basal medium (11875093, ThermoFisher Scientific, Mississauga, ON, Canada) supplemented with 2% B27 without insulin (A18956-01, Gibco, ThermoFisher Scientific, Mississauga, ON, Canada), along with 12 µM CHIR99021 (2520691, Biogems, Westlake Village, CA, USA) for 24 h to indirectly activate the Wnt signalling pathway by inhibiting Glycogen synthase kinase 3 (GSK3). Following this, the cells were treated with 5 µM IWP4 (5214, TOCRIS, Bio-Techne Canada, Toronto, ON, Canada), a Wnt inhibitor, in combination with RPMI 1640 and 2% B27 without insulin. aCMs differentiation was induced by administering 0.75 µM retinoic acid (RA) (R2625-50MG, Sigma-Aldrich, Oakville, ON, Canada), the biologically active form of vitamin A, every 24 h from days 4 to 6. This RA exposure during a specific developmental window converts a subset of cardiovascular mesoderm cells into aCMs. The medium was then switched to RPMI 1640 supplemented with 2% B27 50X with insulin (17504-044, Gibco, ThermoFisher Scientific, Mississauga, ON, Canada), with subsequent medium changes every 2 days. By days 8 to 11, spontaneous beating of hiPSC-aCMs was typically observed. Purification of cardiomyocytes was conducted by introducing sodium L-lactate (71718, Sigma-Aldrich, Oakville, ON, Canada) while omitting glucose from the media. Subsequently, the hiPSC-aCMs underwent a maturation process for 5 weeks in maturation media based on Feyen et al. [33] to improve their electrophysiological characteristics.

### 2.2. Preparation of hiPSC-aCMs for Automated Patch-Clamp Experiments

**Figure 2:**
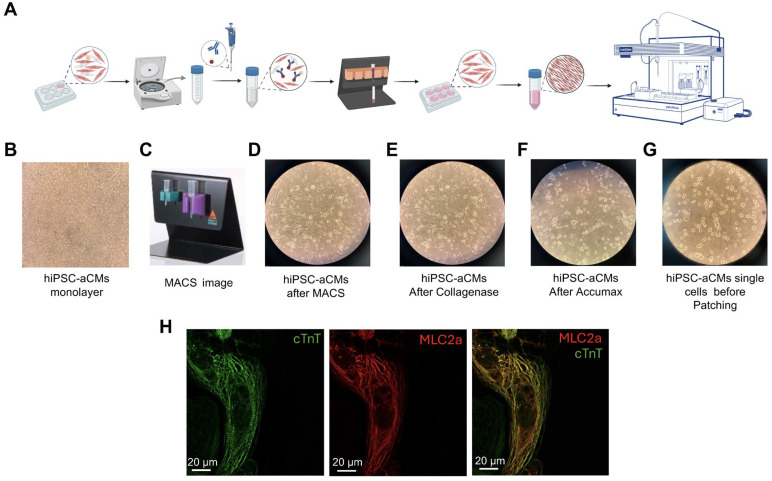
Optimized preparation protocol for hiPSC-aCMs using magnetic-activated cell sorting (MACS^®^). (**A**) Schematic workflow representation of the preparation process of hiPSC-aCMs for electrophysiological analysis using the Patchliner system. (**B**) hiPSC-aCM monolayer (10X objective). (**C**) Magnetic activated cell sorting Miltenyi set up. (**D**) Plated hiPSC-aCMs after purification with MACS^®^ (10X objective). (**E**) hiPSC-aCMs illustration after 30 min of incubation in Collagenase B. (**F**) hiPSC-aCMs illustration after 10 min of incubation in Accumax. (**G**) Resuspended dissociated single atrial cardiomyocytes. (**H**) Fluorescence images captured using a confocal SP8 60X objective showing immunolabeling of cardiac troponin T (cTnT, green), myosin light chain 2a (MLC2a, red), and their overlay image.

To ensure optimal cell membrane quality for subsequent automated patch-clamp experiments, we developed an optimized protocol for hiPSC-aCM preparation (see the workflow shown in Figure 2A). Although monolayers of hiPSC-aCMs (Figure 2B) underwent metabolic selection between days 11 and 15 of differentiation to decrease the number of non-cardiomyocyte cells within the culture, we designed a second purification step using magnetic-activated cell sorting technology (MACS^®^; Miltenyi Biotec, Gaithersburg, MD, USA) (Figure 2C) after 5 weeks of maturation treatment to further purify the atrial cardiomyocytes. A negative selection protocol to target the non-cardiomyocytes was used, which typically allowed us to obtain over 90% hiPSC-aCMs (Figure 2D). After purification, hiPSC-aCMs were seeded at a low density to foster single-beating cells within each well, totaling approximately 600,000 hiPSC-aCMs per well after replating. To confirm that the majority of the low-density hiPSC-CMs exhibit an atrial phenotype, immunocytochemistry was performed using antibodies against cardiac troponin T (cTnT), a general cardiomyocyte marker, and myosin light chain 2a (MLC2a), a marker specific to atrial cardiomyocytes. As shown in Figure 2H, a representative single cardiomyocyte displayed substantial expression and co-localization of MLC2a with cTnT, supporting the identification of the cell as an atrial cardiomyocyte. These cells underwent a recovery period of approximately 2 weeks before dissociation for patch-clamp experiments, so the recordings were obtained from 9-week-old cells.

Following recovery, hiPSC-aCMs were dissociated using a customized protocol utilizing Collagenase B (1 mM) (LS004147, Worthington Biochemical Corporation, Lakewood, NJ, USA) and Accumax^TM^ (07921, STEM CELL Technologies, Vancouver, BC, Canada). hiPSC-aCMs were incubated in Collagenase B for 15–30 min, depending on the maturation stage of the cells (Figure 2E). The more mature the cells, the longer the incubation time that was required. After the suitable incubation time with Collagenase B, the enzyme was removed, and the cells were incubated in Accumax^TM^ as the secondary digestive enzyme. Accumax^TM^ incubation time varied in the 5–15 min range, depending on the aforementioned parameters (Figure 2F). The resulting hiPSC-aCMs were counted and resuspended in a 1:1 mixture of RPMI supplemented with B27 and external solution and then transferred to the Patchliner cell hotel for experimental procedures (Figure 2G).

### 2.3. Automated Patch-Clamp Technique

Electrophysiological recordings were performed using a Patchliner (Nanion Technologies GmbH, Munich, Germany) automated patch-clamp rig. The low-density mature hiPSC-aCMs were freshly isolated with the described optimized dissociation protocol and kept in suspension at 12 °C. The pipette, chip wagon, and measure head temperature were set at 25 °C in all the experiments to ensure consistency. Medium-resistance NPC-16 chips (1.8–3 MΩ) were used in all experiments, as they resulted in a significantly higher catch rate (4–6 cells out of 8) compared to low- and high-resistance chips. A summary table showing the number of cells expressing the current of interest relative to the total number of captured cells is shown in Table 1. Cells that met the criteria of >200 MΩ seal resistance and <10 MΩ series resistance were included in the analysis. In addition, following multiple recordings from both mature and immature cells, a quality-control criterion was established based on the presence of both detectable I_Na_ and I_to_ currents, which are hallmark features of atrial electrophysiology and may indicate a more mature phenotype. Only cells that met these electrophysiological and quality-control criteria were used for subsequent analyses.

#### 2.3.1. Solutions and Specific Protocols Used for Electrophysiological Recordings

Nanion’s standard internal, external, and seal enhancer solutions (whose compositions are specified below) were used to record most of the desired ion currents. Nevertheless, slight adjustments to these solutions were necessary for the accurate measurement of certain currents included in this study. The details of these adjustments and the specific protocols are outlined in the subsections related to each current. Nanion extracellular standard solution for recording Na^+^ and K^+^ currents contained the following (in mM): 140 NaCl, 4 KCl, 1 MgCl_2_, 2 CaCl_2_, 5 D-Glucose monohydrate, and 10 Hepes (pH = 7.4, Osm = 298 mOsmol).

The standard intracellular solution for recording Na^+^ and Ca^2+^ currents contained the following (in mM): 50 CsCl, 10 NaCl, 60 CsF, 20 EGTA, and 10 HEPES (pH 7.2, Osm = 285 mOsmol). The standard intracellular solution for recording K^+^ currents contained the following (in mM): 50 KCl, 10 NaCl, 60 K-Fluoride, 20 EGTA, and 10 Hepes (pH = 7.2, Osm = 285 mOsmol).

The seal enhancer solution contained the following (in mM): 80 NaCl, 3 KCl, 10 MgCl_2_, 35 CaCl_2_, and 10 Hepes (pH = 7.4, Osm = 298 mOsmol).

#### 2.3.2. Measurement of Na^+^ Current

Nanion standard extracellular, intracellular, and seal enhancer solutions were utilized to record I_Na_. However, to achieve improved voltage control in the I_Na_ recordings, experiments were conducted using extracellular solutions with a reduced extracellular sodium concentration ([Na^+^]_o_) of 30% (42 mM) and 10% (14 mM) relative to the standard solution. An I-V step protocol was used to study the current-voltage relationship of I_Na_. Cells were held at a holding potential of −100 mV, and 20 ms depolarizing voltage steps were applied from −80 mV to +40 mV in 10 mV increments.

#### 2.3.3. Measurement of L-Type Calcium Current

The extracellular solution used to record I_CaL_ was previously reported by Li et al. [26] and contained (in mM) 150 TEA-Cl, 10 D-glucose monohydrate, 10 HEPES, 1 MgCl_2_, and 2 CaCl_2_ (pH = 7.4). Nanion intracellular and seal enhancer standard solutions were used. On the day of the experiment, 0.3 mM Na_3_GTP, 5 mM ATP (Mg salt), and 5 mM BAPTA were freshly added to minimize current rundown. An I-V step protocol was used to determine the voltage-dependency of the I_CaL_. Cells were held at a holding potential of −80 mV, followed by a 100 ms pre-pulse step to −40 mV to inactivate Na^+^ channels and subsequent 200 ms depolarizing voltage steps from −40 to +40 mV in 10 mV increments. Steady-state I_CaL_ was recorded using a pacing interval of 2 s. Currents were evoked by applying a 50 ms pre-pulse from −80 mV to −40 mV, followed by a 200 ms depolarizing step to 0 mV. The I_CaL_ density was measured as the peak current normalized to cell capacitance. The recordings were obtained in control conditions and after incubation with 1 µM BAY-K (an L-type calcium channel-specific activator) and 1 µM nifedipine (a L-type calcium channel blocker).

#### 2.3.4. Measurement of Major Atrial Potassium Repolarizing Currents

Nanion Standard extracellular, intracellular, and seal enhancer solutions were used to record the major atrial K^+^ repolarizing currents. The voltage-dependence of the K^+^ current was determined using an I-V step protocol. Cells were held at a holding potential of −80 mV, and 500 ms depolarizing voltage steps were applied from −40 to +50 mV in 10 mV increments, following a 100 ms step to −40 mV to inactivate Na^+^ channels. The protocol was performed under control conditions, and after incubation with the voltage-gated potassium channel inhibitor 4-aminopyridine (4-AP, 2 mM). The peak transient outward current (I_peak_) was measured as the difference between the peak current and the current at 200 ms from the beginning of the depolarization pulses. The sustained current (I_sus_) was measured as the current at 200 ms from the beginning of the depolarization pulses. The 4-AP-sensitive I_peak_ includes contributions from both I_to_ and I_Kur_, whereas the I_sus_ primarily reflects I_Kur_ amplitude, since I_to_ and I_Kr_ are negligible at the time the I_sus_ is being calculated, as previously demonstrated [34].

#### 2.3.5. Measurement of Small-Conductance Calcium-Activated Potassium Current

Nanion standard extracellular, intracellular, and seal enhancer solutions were used to record the small-conductance calcium-activated potassium current (I_SK_). An I-V ramp voltage protocol was used to study the current-voltage relationship of I_SK_. The I-V ramp protocol was conducted over a voltage range of −80 mV to +60 mV at a rate of 28 mV/s. Current measurements were recorded in control conditions and after adding 100 nM of apamin, a specific SK channel inhibitor. The subtracted apamin-sensitive current was taken as I_SK_.

#### 2.3.6. Measurement of Funny Current

Nanion standard extracellular, intracellular, and seal enhancer solutions were employed to record funny currents (I_f_) resulting from hyperpolarization-activated cyclic nucleotide-gated (HCN) channels. However, additional experiments were performed by increasing the extracellular K^+^ concentration to 30 mM. The I_f_ was activated from a holding potential of −40 mV by applying a series of 10 mV hyperpolarizing voltage test steps of 1s from −40 mV to −110 mV, followed by a depolarization step of 250 ms at +30 mV. The I-V relationship was determined by calculating the difference between the initial and final amplitude for the slowly activating current for each test voltage pulse. To investigate the effect of I_f_ inhibition, the specific inhibitor ivabradine (1 μM) was employed. Time constants of activation (τ_act_) were obtained by fitting the current traces recorded during the voltage steps from −110 mV to −90 mV in experiments performed under control conditions with 30 mM extracellular K^+^ using a single exponential function. Values of time constants were used in this analysis from cells whose R^2^ values from the fitting were >0.9 for the three current traces at −90 mV, −100 mV, and −110 mV.

### 2.4. Data Analysis

#### 2.4.1. Statistical Analysis

Quantitative variables are represented as the mean ± standard error of the mean (SEM), unless otherwise specified. As the variables were normally distributed, one-way or two-way ANOVA were conducted, followed by Tukey’s post hoc analysis. All statistical analyses were executed using GraphPad Prism 9.5.1. The specific tests employed are detailed in each figure’s caption.

#### 2.4.2. Mathematical Analysis

The sodium equilibrium potential (E_Na_) at 25 °C was calculated using the Nernst Equation (1).

E_Na_ = (RT/zF) ln([Na^+^]_o_/[Na^+^]_i_) (1)

where R is the universal gas constant (8.3114 J), T is the temperature in degrees Kelvin, z is the valence of the ion, F is the Faraday constant (96.483 C/mol), and [Na^+^]_o_ and [Na^+^]_i_ are the concentrations of Na^+^ outside and inside, respectively.

The curve fitting for the Na^+^ conductance/maximal Na^+^ conductance (G/G_Max_)-voltage relation was obtained using Equation (2) [35]:

G_Na_ = G_Max_/[1 + exp[(V − V_half_)/s] (2)

## 3. Results

### 3.1. Sodium Current Characterization in hiPSC-aCMs

**Figure 3:**
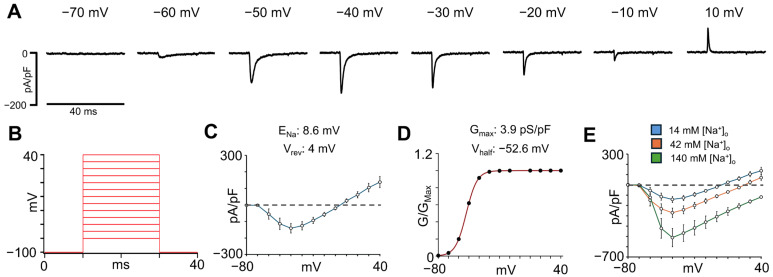
Sodium current characterization in hiPSC-aCMs using a [Na^+^]_o_ concentration of 14 mM. (**A**) Representative I_Na_ traces at voltages between −70 and +10 mV normalized to membrane capacitance. (**B**) Voltage protocol used to obtain the current-voltage (I-V) relationship for the I_Na_. (**C**) I-V relationship determined in single hiPSC-aCMs (*n* = 11). The equilibrium potential from Na^+^ (E_Na_) and the reversal potential (V_rev_) calculated from the I-V relationship are shown above. (**D**) Normalized conductance (G/G_Max_)-voltage curve of the I_Na_ from the same hiPSC-aCMs used for the I-V relationship. The equation specified in the Methods section was used to fit this curve. The calculated values for the maximum conductance (G_Max_) and the half-activation voltage (V_half_) are given above. (**E**) I-V relationships recorded in the same hiPSC-aCMs using 14 mM, 42 mM, and 140 mM [Na^+^]_o_.

The I_Na_ plays a critical role in the initiation and propagation of the cardiac action potential (AP), determining the upstroke velocity and overall excitability of atrial cardiomyocytes. To characterize the I_Na_ density in isolated single hiPSC-aCMs, the voltage-clamp protocol shown in Figure 3B was used. As illustrated in the representative I_Na_ recordings normalized to membrane capacitance (pA/pF) in Figure 3A, hiPSC-aCMs displayed robust inward I_Na_ that activated upon depolarization. The current-voltage (I-V) relationship revealed that I_Na_ increased with depolarization and reached a maximal peak amplitude of −141 ± 29 pA/pF (*n* = 11) at −40 mV under 14 mM [Na]_o_ (Figure 3C). The E_Na_, calculated using Equation (1) (see Methods), was 8.6 mV, which is consistent with the reversal potential (V_rev_) obtained from the I-V relationship (4 mV), confirming accurate voltage control. From the same recordings, the conductance (G/G_Max_)-voltage relationship (Figure 3D) was calculated using the mean peak value at each potential. The curve was fitted using Equation (2), specified in the Methods section. The mean V_half_ was −52 ± 4 mV, and the G_Max_ before normalization was 3.9 ± 0.3 pS/pF. Importantly, the voltage of peak I_Na_ occurred at the same voltage when different [Na^+^]_o_ were used (Figure 3E).

### 3.2. L-Type Calcium Current Characterization in hiPSC-aCMs

**Figure 4:**
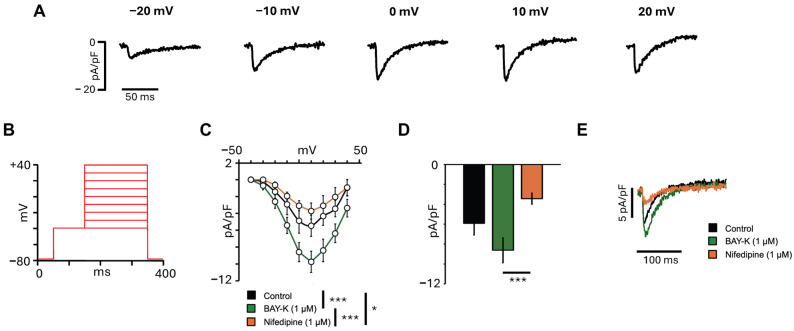
L-type calcium current characterization in hiPSC-aCMs. (**A**) Representative I_CaL_ traces from a cell under control conditions at different voltages. (**B**) Voltage protocol used to obtain the current-voltage relationship for the I_CaL_. (**C**) I-V relationship recorded in the same hiPSC-aCMs in control and after the addition of the activator BAY-K (1 µM) and the inhibitor nifedipine (1 µM) (*n* = 9). Statistical significance was determined using a repeated-measures two-way ANOVA followed by Tukey’s HSD post hoc. (**D**) Mean I_CaL_ amplitude recorded at 0 mV in control and after the addition of BAY-K (1 µM) and subsequent addition of nifedipine (1 µM) (*n* = 9). Statistical significance was determined using a one-way ANOVA followed by Tukey’s HSD post hoc. (**E**) Representative I_CaL_ traces from the same cell recorded at 0 mV in control, after addition of BAY-K (1 µM) and subsequent addition of nifedipine (1 µM). The significant differences are indicated with the following: * *p* < 0.05; *** *p* < 0.001.

The I_CaL_ is a key inward current that contributes to the plateau phase of the cardiac AP and is essential for excitation-contraction coupling in atrial cardiomyocytes. To characterize I_CaL_ density in hiPSCs-aCMs, the voltage protocol shown in Figure 4B was used. As illustrated in the representative traces of Figure 4A, depolarizing voltage steps elicited inward calcium currents that reached the peak at +10 mV, consistent with the activation profile of I_CaL_. The I-V relationship (Figure 4C) revealed that the mean current density reached the peak at +10 mV and was significantly modulated by pharmacological agents, although no differences in the shape of the curve were observed. The mean current density recorded at 0 mV (Figure 4D) resulted in a 1.4-fold increase after incubation with BAY-K (1 µM), while subsequent treatment with nifedipine (1 µM) led to a significant 60.2% reduction (*p* = 0.0008). Representative I_CaL_ traces for each condition are shown in Figure 4E.

### 3.3. Characterization of the Major Atrial K^+^ Repolarizing Currents in hiPSC-aCMs

**Figure 5:**
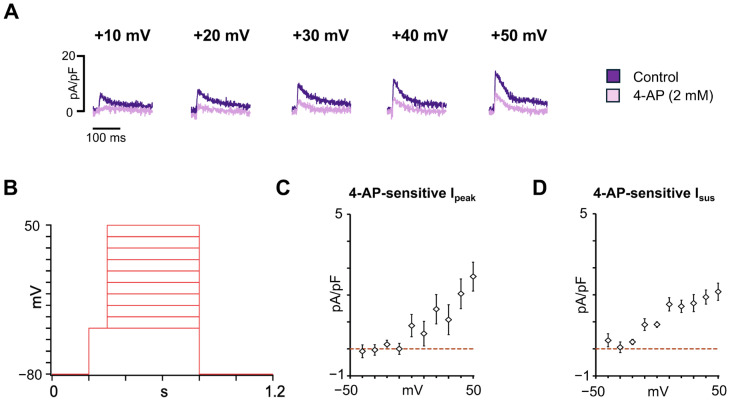
Major atrial potassium repolarizing currents characterization in hiPSC-aCMs. (**A**) Representative traces recorded at different voltages before (control) and after the addition of 4-AP (2 mM). (**B**) Voltage protocol used to obtain the current-voltage relationship of the major atrial K^+^ repolarizing currents. (**C**) Current-voltage (I-V) relationship for the 4-AP-sensitive peak transient outward current (I_peak_) (*n* = 6). (**D**) I-V relationship for the 4-AP-sensitive sustained current (I_sus_) (*n* = 6). Dashed lines in panels C and D indicate the zero current level.

The I_to_ and I_Kur_ are 4-AP-sensitive and represent key atrial repolarizing currents that shape the early and late phases of the atrial AP, contributing to the electrophysiological distinction between atrial and ventricular cardiomyocytes. To characterize these repolarizing K^+^ currents in hiPSC-aCMs, the voltage-clamp protocol shown in Figure 5B was used. Figure 5A shows representative current traces recorded at different depolarizing voltages before and after the addition of 2 mM 4-AP, which reduced both the I_peak_ and I_sus_. The I-V relationship of the 4-AP-sensitive I_peak_ is presented in Figure 5C and was 2.7 ± 0.5 pA/pF (*n* = 6) at +50 mV. The I-V relationship for 4-AP-sensitive I_sus_, representing I_Kur_, is shown in Figure 5D and was 2.1 ± 0.3 pA/pF (*n* = 6) at +50 mV. Together, these findings demonstrate that hiPSC-aCMs express functional 4-AP-sensitive I_to_ and I_Kur_ currents, supporting their electrophysiological identity as atrial-like cardiomyocytes.

### 3.4. Apamin-Sensitive SK Current Characterization in hiPSC-aCMs

**Figure 6:**
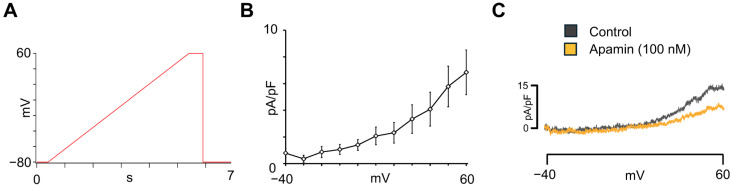
Small-conductance Ca^2+^-activated K^+^-current characterization in hiPSC-aCMs. (**A**) Voltage ramp protocol used to obtain the I-V relationship for the apamin-sensitive I_SK_. (**B**) Voltage-dependence of apamin-sensitive I_SK_ obtained from hiPSC-aCMs (*n* = 16). (**C**) Representative current recordings before (control) and after the addition of apamin (100 nM).

SK channels are important determinants of the atrial electrophysiological phenotype, as they contribute to repolarization and calcium-dependent regulation of AP duration (APD), and have recently been proposed as potential therapeutic targets for AF. To our knowledge, there is no published literature on the characterization of apamin-sensitive SK current in hiPSC-aCMs using automated patch-clamp technology. To investigate the presence of SK currents in hiPSC-aCMs, apamin-sensitive SK currents were recorded. The voltage-ramp protocol used for these recordings is shown in Figure 6A. The I-V relationship of the apamin-sensitive current, obtained by subtracting current traces recorded in the presence of apamin from those under control conditions, is presented in Figure 6B (*n* = 16). As shown in the representative traces in Figure 6C, a depolarizing voltage ramp elicited outward currents that were markedly reduced after the addition of 100 nM apamin. These results confirmed the functional expression of SK channels in hiPSC-aCMs.

### 3.5. Funny Current Characterization in hiPSC-aCMs

The I_f_ contributes to the diastolic depolarization that controls spontaneous rhythm generation in the sinoatrial node (SAN), and alterations in HCN expression and function have been associated with atrial arrhythmias [36,37]. To characterize the I_f_ current in hiPSC-aCMs, we used an external solution (see Methods) containing 30 mM [K^+^]_o_, which produced an expected increase in I_f_. Raising extracellular potassium has been shown to increase I_f_ amplitude in Purkinje fibres [38] and sinoatrial pacemaker cells [39], as well as in heterologous cells that express mammalian HCN forms [38,40,41]. To help identify the I_f_, we used 1 μM ivabradine. This drug has been reported to inhibit I_f_ of the rabbit SAN with an EC_50_ of 1.5 mM [42].

**Figure 7:**
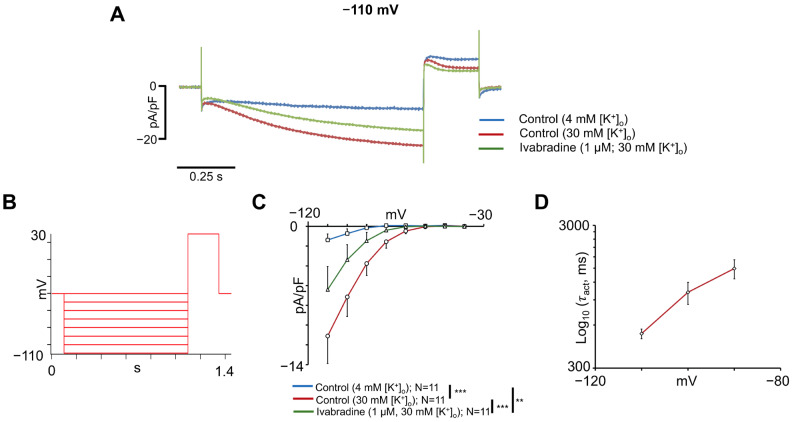
Funny current characterization in hiPSC-aCMs. (**A**) Representative I_f_ traces recorded at −110 mV with 4 mM [K^+^]_o_, 30 mM [K^+^]_o_, and 30 mM [K^+^]_o_ plus 1 μM ivabradine. (**B**) Voltage protocol used to obtain the current-voltage relationship of I_f_. (**C**) Current-voltage (I-V) relationship for I_f_ with 4 mM [K^+^]_o_, 30 mM [K^+^]_o_, and 30 mM [K^+^]_o_ plus 1 µM ivabradine. Statistical significance in panel (**C**) was determined using a repeated-measures two-way ANOVA followed by Tukey’s HSD post hoc test. Multiple comparisons between conditions have been performed and are indicated with **: *p* < 0.01; ***: *p* < 0.001. (**D**) Voltage-dependent activation time constants (τ_act_) obtained as described in Methods section for I_f_ current elicited at voltage steps from −110 mV to −90 mV in experiments performed under control conditions with 30 mM [K^+^]_o_. τ_act_ is in logarithmic scale, and *n* = 5–7 cells per voltage step.

Figure 7A shows representative traces recorded at −110 mV with 4 mM [K^+^]_o_, 30 mM [K^+^]_o_, and 30 mM [K^+^]_o_ plus 1 µM ivabradine. The voltage protocol used to record the I-V relationships is shown in Figure 7B and consisted of a holding voltage of −40 mV and a series of subsequent hyperpolarizing test voltages. Statistical analysis of I-V relationships (Figure 7C) showed significant differences between the conditions (*p* = 0.0028). At −110 mV, the mean current density increased from −1.4 ± 0.6 pA/pF under 4 mM [K^+^]_o_ to −11.1 ± 2.8 pA/pF with 30 mM [K^+^]_o_ (*n* = 11, *p* < 0.01), and decreased to −6.4 ± 2.3 pA/pF after the addition of 1 μM ivabradine (*n* = 11, *p* < 0.01). Analysis of the time constants of current activation (Figure 7D) showed a voltage-dependent slowing of activation at less negative potentials, consistent with the gating properties of I_f_ channels. The observed voltage-dependence, sensitivity to ivabradine, and modulation by extracellular K^+^ confirm that hiPSC-aCMs express a functional I_f_ with properties resembling those reported in native cardiac pacemaker cells.

## 4. Discussion

Alterations in the ion channels involved in the cardiac AP and/or Ca^2+^ handling proteins have been shown to underlie atrial electrical remodelling and ectopic activity, contributing to the appearance of atrial arrhythmias such as AF. Accordingly, several studies have linked genetic variants in genes encoding sodium (*SCN5A*, I_Na_) [43], potassium (*KCND3*,I_to_; *KCNA5*, I_Kur_; *KCNN2*, I_SK2_; *KCNN3*, I_SK3_) [44,45,46,47], calcium (*CACNB2* and *CACNA2D4*, I_CaL_) [48], and *HCN4* (I_f_) [49] channels with AF. Alterations in the function of these channels can modify AP properties, conduction velocity, and pacemaking frequency, thereby increasing susceptibility to AF. Thus, functional characterization of these variants in relevant human atrial tissue is essential.

Electrophysiological techniques, such as manual patch-clamp, have long been used to characterize ion channel functionality in cardiomyocytes derived from hiPSCs, human tissue, or animal models. However, because the manual patch-clamp technique is very labour-intensive and low-throughput, significant efforts have been made in the last decade to improve the automated patch-clamp systems [24,26,50]. One of the most critical factors for using automated patch-clamp systems is the preparation of high-quality unicellular cell suspensions. Here, we present an optimized protocol (Figure 2) to maintain membrane integrity while minimizing enzymatic and mechanical membrane damage and cytotoxicity, thereby enabling reliable recordings of atrial currents using an automated patch-clamp system. Thus, our protocol using the replating of hiPSC-aCMs at low density as single cells after purification using the MACS^®^ technology facilitates a more efficient and gentle preparation process that allows the cells to recover from the replating procedure while maintaining membrane integrity.

Another challenge of using hiPSC-CMs has been their structural, electrophysiological, calcium handling, and metabolic immaturity compared to adult cardiomyocytes [51]. In this context, previous studies on hiPSC-CMs employing automated patch-clamp technology reported membrane capacitance values from 17 to 40 pF [28], whereas the cells obtained with our optimized protocol yielded values ranging from 11 to 103 pF (mean of 37.6 pF), closely approximating those measured in native human atrial myocytes [52]. These findings are consistent with Li et al. [26], who also reported improved maturation and I_Na_ density using refined dissociation procedures. Thus, the optimized protocol presented in this study enhances both electrophysiological stability and morphological maturation of hiPSC-aCMs, providing a physiologically relevant human atrial model.

Among the characterized ionic currents, the I_Na_ is fundamental for the rapid depolarization phase of the cardiac AP and determines conduction velocity and excitability in atrial tissue. Altered I_Na_ can slow conduction and facilitate re-entry, contributing to AF substrates. The *SCN5* gene encodes the α subunit of the voltage-gated sodium channel (Na_v_1.5) and plays an essential role in the generation and propagation of the cardiac AP [53]. The I_Na_ peak density obtained in this study (−141 ± 29 pA/pF at −40 mV) aligns with manual patch-clamp data from native atrial myocytes of several species, including rabbit [54], dog [55], rat [56], and human [57]. Differences across studies may be attributed to differences in [Na^+^]_o_ and β-subunit expression, notably *SCN1B* and *SCN3B,* which are known to regulate Na_v_1.5 expression and modulate current density [58,59]. In addition, reduced connexin 43 (*GJA1*) expression has been linked to decreased I_Na_ [60,61].

Our findings align with the study conducted by Li et al. [26] in hiPSC-CMs, reporting a peak I_Na_ of −187 pA/pF, which was two-fold greater than that reported in prior investigations [28], which they attributed to enhanced cellular maturation or a gentler cell dissociation process [26]. Together, these findings confirm that our protocol preserves functional sodium channels with densities comparable to native atrial cells.

The I_CaL_ sustains the AP plateau phase and regulates excitation-contraction coupling. Alterations in I_CaL_ can promote triggered activity, delayed afterdepolarizations, and abnormal calcium cycling, all of them key features in AF pathophysiology. The mean I_CaL_ density recorded at 0 mV in this study was −5.9 ± 1.2 pA/pF, which aligns with previous studies in native human atrial myocytes using manual patch-clamp [7,60,62] and in native swine atrial myocytes using automated patch-clamp [63]. According to previous dose-response studies, a concentration of 1 μM nifedipine was used in this study to observe the maximum blocking effect [64,65]. However, a complete block of I_CaL_ was not achieved in this study, possibly due to the recording sequence involving the activator BAY-K followed by the blocker. On the other hand, few studies have assessed I_CaL_ in hiPSC-CMs, and reported values show considerable variability. Notably, approximately 10% of monolayer-derived hiPSC-CMs have been reported to lack detectable I_CaL_, potentially reflecting differences in maturation or differentiation [60,66]. Thus, this study provides standardized protocols and recording conditions for I_CaL_ in hiPSC-aCMs, facilitating comparison with I_CaL_ measured in native human atrial cardiomyocytes.

Voltage-gated potassium (K_v_) channels play a pivotal role in cardiac repolarization and myocardial excitability. Among these, the I_to_, comprising fast (I_to,f_, primarily mediated by K_v_4.3 and K_v_4.2) and slow components (I_to,s_, attributed to Kv1.4) [67,68], contributes to early repolarization [69] and exhibits marked differences between atrial and ventricular cells [70]. In addition, I_Kur_ is mediated by K_v_1.5, which is highly expressed in human atria [71], and also participates in early atrial repolarization [72]. Alterations in both I_to_ and I_Kur_ have been implicated in arrhythmogenic disorders such as Brugada syndrome, early repolarization syndrome [73], and AF [34,46,47]. In this study, the I_peak_ recorded in hiPSC-aCMs displayed a rapidly activating and inactivating profile, consistent with I_to_ properties previously reported in native human atrial cardiomyocytes [34,74]. On the other hand, the I_sus_ recorded at +50 mV in this study, which is mainly attributed to I_Kur_, aligns with previous reports on hiPSC-aCMs [32]. However, there is variability in I_to_ and I_Kur_ between other reports on hiPSC-CMs [31,75,76], which may be attributed to the maturity state of hiPSC-aCMs [77], variability in differentiation methods, and experimental conditions such as temperature or ionic composition of the bath solutions. Electrical heterogeneity has also been reported in isolated native human atrial myocytes [34], in which cells from the sinus rhythm and AF displayed differences in the K^+^ plateau phase (I_to_-predominant, I_sus_-predominant, or intermediate pattern). Despite these discrepancies, the results obtained from the hiPSC-aCMs in this study reproduce atrial-specific repolarization, supporting their use as a platform to investigate the molecular mechanisms of *KCND3* or *KCNA5*-associated AF variants and to evaluate drugs modulating repolarization.

Atrial repolarization is also modulated by SK channels, which have emerged as a novel therapeutic target for AF [78,79,80]. Previous studies using hiPSC-CMs lacking atrial specification reported that apamin, an SK-channel blocker, failed to prolong repolarization [81]. However, a recent study compared the effects of SK channel inhibition using patch-clamp technique in isolated hiPSC-aCMs and monolayer cultures of hiPSC-aCMs [82]. While only a minority of isolated hiPSC-aCMs responded to the application of the SK channel blocker UCL1684 (100 nM), all hiPSC-aCMs monolayers demonstrated a prolongation of AP duration at 50% repolarization (APD_50_) following UCL1684 treatment. The amplitude of the apamin-sensitive SK current reported in Figure 6 of this work is consistent with that observed in native human atrial myocytes using manual patch-clamp technique [83,84,85]. These results demonstrate that the hiPSC-aCMs reproduce atrial-specific repolarization features and provide a human model for testing SK-targeted therapies.

The I_f_ plays a crucial role in diastolic repolarization and cardiac pacemaking, and numerous studies have linked alterations in HCN channel expression or function with sinus node dysfunction, AF, and cardiomyopathies [86,87,88,89,90,91,92]. Due to the limited availability of human hearts and the anatomic inaccessibility of the SAN, obtaining human SAN biopsies is unfeasible. Consequently, most investigations into the physiological and pathological roles of I_f_ have been conducted in rabbit and murine SAN cells [42,91]. hiPSC-aCMs offer a human-based alternative since some display high I_f_ and low I_K1_, which are both hallmark features of the sinoatrial and atrioventricular pacemaker cells [29,39,93,94]. In the present study, the mean I_f_ density recorded at −110 mV was lower than values reported in previous studies involving human SAN diseased cells [90], hiPSCs-CMs[60,95,96], and human embryonic stem cell-derived cardiomyocytes [97], but showed the expected dependence of I_f_ on extracellular K^+^ concentration, even exceeding responses reported in rabbit SAN cells [39]. Although evidence suggests that HCN4 is the major form found in the hearts of mammals [92,98], the relative expression of HCN isoforms may vary depending on cardiomyocyte type, functional state, and maturation stage [99,100,101]. Previous studies have shown that the different HCN isoforms exhibit distinct activation kinetics [102,103,104], and the time activation constants of this study may be in the range of HCN2, HCN3, and HCN4 activation. However, the variability of I_f_ current density and activation kinetics across studies may be attributed to differences in experimental conditions and models, HCN isoform expression, or cell maturity. Nevertheless, cell-to-cell variation in I_f_ density and expression that was observed in the hiPSCs-aCMs of this study has also been noted in a previous report by our group in stem cell-derived atrial cells from mice [105]. In our previous report of stem cell-derived atrial cells from mice, higher levels of I_f_ correlated with faster beating rates [105], and this has also been observed in other animal pacemaker tissues [39,93]. Thus, it seems possible that there are hiPSC-CMs that possess high levels of I_f_, beat at the highest rate, and act as primary pacemakers. These results reinforce the potential of hiPSC-aCMs for studying atrial pacemaking and AF-associated *HCN4* variants.

Overall, this study advances the use of hiPSC-aCMs as a physiologically relevant platform for investigating human atrial electrophysiology and arrhythmogenesis. By enabling stable and reproducible recordings of multiple key ionic currents using automated patch-clamp technology, our work overcomes limitations of conventional manual approaches. The electrophysiological properties observed resemble those of native atrial cardiomyocytes, establishing a strong methodological foundation for mechanistic investigations into atrial arrhythmogenesis and the validation of atrial-selective pharmacological targets.

Although hiPSC-aCMs represent a promising model for investigating atrial arrhythmias and addressing some of the challenges of using animal models, other limitations should be considered. First, it is known that the maturation process undergone by the hiPSC-aCMs used in this study can result in a heterogeneous maturation state, which can influence ion channel expression and contribute to certain variability in the data. This may be particularly relevant when comparing results from hiPSCs-CMs and native cardiomyocytes obtained from animals or human tissue. Furthermore, as patch-clamp experiments are conducted in single cells, future integration with complementary techniques available in our laboratory (such as high-speed optical mapping or micro-optical mapping) in 2D monolayers and 3D structures would help understand the electrophysiological behaviour of cells within a tissue context. Finally, more detailed molecular characterization of the atrial phenotype will be an important goal for future studies.

## 5. Conclusions and Clinical Relevance

This study elucidates the feasibility of using automated patch-clamp technology to study the functional impact of genetic risk variants affecting the function of ion channels addressed in this study. Furthermore, it underscores the importance of using an optimized cell dissociation protocol to ensure the quality of the experiments, which has allowed us to record major atrial ionic currents such as I_Na_, I_CaL,_ I_to,_ I_Kur_, I_SK_, and I_f_. This approach provides a powerful platform for the functional characterization of ion channels, enabling the detection of electrophysiological dysfunction caused by pathogenic genetic variants. In conclusion, employing automated patch-clamp technology in hiPSC-aCMs models is a valuable and efficient tool for drug screening and precision medicine that can potentially facilitate the development of targeted therapies for atrial arrhythmias and ion channel-related disorders.

## Figures and Tables

**Table 1:** Electrophysiological parameters for each recorded current. The table shows the % of cells expressing the currents and the electrophysiological parameters recorded for each current. The % of cells expressing the current is presented as the proportion of cells exhibiting the current of interest relative to the total number of captured cells. Only cells meeting the criteria mentioned above were used for subsequent analyses. The electrophysiological parameters are expressed as the mean ± SEM, with the number of cells contributing to each parameter’s mean indicated in parentheses.

Parameters (N)	I_Na_	I_CaL_	I_to/_I_Kur_	I_SK_	I_f_
Cells expressing current, %	14/22, 64%	12/25, 48%	10/22, 45%	23/40, 58%	15/33, 45%
Seal resistance, GΩ	0.5 ± 0.1 (11)	3.7 ± 1.5 (9)	1.0 ± 0.4 (6)	0.9 ± 0.2 (16)	0.4 ± 0.2 (11)
Series resistance, MΩ	5.5 ± 0.5 (11)	5.8 ± 1.0 (9)	6.2 ± 1.3 (6)	6.4 ± 0.5 (16)	6.5 ± 0.7 (11)
Cell capacitance, pF	35.8 ± 0.5 (11)	32.8 ± 6.0 (9)	36.4 ± 6.3 (6)	36.6 ± 5.0 (16)	55.2 ± 9.7 (11)

## Data Availability

The data supporting the findings of this study are available from the corresponding author upon reasonable request.

## Abbreviations

The following abbreviations are used in this manuscript:

4-AP	4-aminopyridine
AF	Atrial fibrillation
AP	Action potential
APD	Action potential duration
cTnT	Cardiac troponin T
CPVT	Catecholaminergic polymorphic ventricular tachycardia
DAD	Delayed afterdepolarization
E_Na_	Sodium equilibrium potential
HCN	Hyperpolarization-activated cyclic nucleotide-gated
hiPSC-aCMs	Human-induced pluripotent stem cell-derived atrial cardiomyocytes
hiPSC-CMs	Human-induced pluripotent stem cell-derived cardiomyocytes
hiPSC-vCMs	Human-induced pluripotent stem cell-derived ventricular cardiomyocytes
I_CaL_	L-type calcium current
I_f_	Funny current
I_Kur_	Ultrarapid component of the delayed rectifier current
I_Na_	Sodium current
I_SK_	Small-conductance calcium-activated potassium current
I_to_	Transient outward potassium current
LQTS	Long QT syndrome
MACS	Magnetic-activated cell sorting
MLC2a	Myosin light chain 2a
SAN	Sinoatrial node
V_rev_	Reversal potential
